# Molecular characterization of *B. anthracis* isolates from the anthrax outbreak among cattle in Karnataka, India

**DOI:** 10.1186/s12866-020-01917-1

**Published:** 2020-07-31

**Authors:** Akanxa Roonie, Saugata Majumder, Joseph J. Kingston, Manmohan Parida

**Affiliations:** Microbiology Division, Defence Food Research Laboratory, Siddartha Nagar, Mysore, Karnataka 570011 India

**Keywords:** *B. anthracis*, 16S rDNA, *Pag*, canSNP, Anthrax detection, Outbreak

## Abstract

**Background:**

Anthrax, a zoonotic disease is caused by the Gram positive bacterium *Bacillus anthracis*. During January 2013, an anthrax outbreak among cattle was reported in Gundlupet Taluk, neighboring Bandipur National Park and tiger reserve, India. The present study aims at the molecular identification and characterization of 12 *B. anthracis* isolates from this outbreak by 16S rRNA gene sequencing, screening *B. anthracis* specific prophages and chromosomal markers, protective antigen (*pag*) gene and canonical single nucleotide polymorphism (canSNP) analysis to subtype the isolates into one of the twelve globally identified clonal sub-lineages of *B. anthracis*.

**Results:**

These isolates had identical 16S rDNA nucleotide sequences with *B. anthracis* specific dual peaks showing mixed base pair R (G/A) at position 1139 with visual inspection while the automated basecaller software indicated a G. Alternatively the nucleotide A at 1146 position was indicative of the 16S rDNA type 7. Multiple sequence alignment with additional 170 (16S rDNA) sequences of *B. cereus* sensu *lato* group from GenBank database revealed 28 new 16S types in addition to eleven 16S types reported earlier. The twelve *B. anthracis* isolates were found to harbor the four *B. anthracis* specific prophages (lambdaBa01, lambdaBa02, lambdaBa03, and lambdaBa04) along with its four specific loci markers (dhp 61.183, dhp 77.002, dhp 73.019, and dhp 73.017). The *pag* gene sequencing identified the isolates as protective antigen (PA) genotype I with phenylalanine-proline-alanine phenotype (FPA phenotype). However, sequence clustering with additional 34 *pag* sequences from GenBank revealed two additional missense mutations at nucleotide positions 196 bp and 869 bp of the 2294 bp *pag* sequence among the 5 *B. cereus* strains with pXO1 like plasmids. The canSNP analysis showed that the isolates belong to A.Br.Aust94 sub-lineage that is distributed geographically in countries of Asia, Africa, Europe and Australia.

**Conclusions:**

The analysis of 16S rDNA sequences reiterated the earlier findings that visual inspection of electropherogram for position 1139 having nucleotide R could be used for *B. anthracis* identification and not the consensus sequence from base caller. The canSNP results indicated that the anthrax outbreak among cattle was caused by *B. anthracis* of A.Br.Aust94 sub-lineage.

## Background

*Bacillus anthracis*, the Gram positive, spore forming bacteria is an etiological agent of anthrax and belongs to a monophyletic lineage of *Bacillus cereus* sensu *lato* group that currently includes 21 published species [1]. *B. anthracis*, being genetically closely related to *B. cereus* and *B. thuringiensis* is believed to have diverged from its *B. cereus* ancestor due to the evolutionary acquisition of two virulence plasmids, pXO1 and pXO2 [2] and often considered as a single species [3]. The protective antigen (*pag)* and capsule (*cap)* genes, located on these plasmids are used as molecular markers for the routine identification of *B. anthracis*. However, occurrence of *B. cereus* and *B. thuringiensis* strains with either or both of these plasmids pose challenges in unambiguous identification of the species [4]. The 16S rDNA gene sequences of *B. cereus* group exhibit a relatively high level of sequence similarity (> 99%) but differ by 13 nucleotide positions that were utilized to identify 16S rDNA types associated with *B. anthracis* [5]. The 16S rDNA gene sequence classified most *B. anthracis* strains in 16S type 6; however, some were reported in type 7 with *B. cereus* strains. Later it was reported that the mixed base pair R (G/A) at position 1139 in *B. anthracis* 16S rRNA gene owing to the presence of multiple rRNA operons in the genome could be used for its differentiation from other closely related *B. cereus* group species [6]. Further, *B. anthracis* specific DNA signature sequences (loci A, C, D, and E) and four prophages (lambdaBa01, lambdaBa02, lambdaBa03, lambdaBa04) located in *B. anthracis* chromosome are also used for definitive discrimination of *B. anthracis* from other *B. cereus* group strains [7, 8]. A defined set of 13 canSNPs from different loci in the genome has been used for molecular typing to determine the phylogenetic position and distinguish geographically separated *B. anthracis* isolates owing to their high level of genetic resolution and discriminatory power [9]. The distribution of these canSNPs identified 12 sub-lineages from the *B. anthracis* clonal lineages A, B and C representing different geographical locations [9].

India hosts the world’s largest livestock population where anthrax is endemic with the highest incidence from the southern part of the country [10, 11]. An epidemiological study by National Animal Disease Referral Expert System (NADRES), India describes anthrax as the third important zoonotic disease in the subcontinent [12]. In these endemic regions, anthrax has also been reported in the livestock handlers [13]. To address the same concern, the National Centre for Disease Control (NCDC), India have been taking efforts for strengthening surveillance, early diagnosis and, effective timely containment of this zoonotic disease [14]. In addition, NCDC along with World Health Organisation (WHO) has also developed training programs for veterinary and medical professionals to control the spread of anthrax [15]. The anthrax vaccine (anthrax spore vaccine) produced in India has been subsidized by the government and is distributed at zero cost to the farmers in the endemic regions through veterinary officials [16].

Recent countrywide surveillance jointly conducted by National Centre for Disease Control (NCDC), Indian Council of Agricultural Research - National Institute of Veterinary Epidemiology and Disease Informatics (ICAR-NIVEDI) and Centers for Disease Control and Prevention (CDC) confirmed anthrax outbreaks in the regions of Odisha and Karnataka [17]. Anthrax associated with infected livestock has later progressed into human cutaneous anthrax due to close proximity with animals. Earlier, human anthrax has been reported due to vector-borne transmission from infected cattle, goat, sheep and buffalo due to handling of infected livestock, slaughtering and even after consumption in few reported cases [18–21]. These data confirm the endemicity of anthrax in the southern states of the subcontinent, that is, Andhra Pradesh, Karnataka and Tamil Nadu [11]. Previously, twelve *B. anthracis* isolates harboring both *pag* and *cap* genes were isolated from an anthrax outbreak among cattle in Gundlupet Taluk neighboring Bandipur National Park, and tiger reserve, India during January 2013 [22]. The present study aimed to identify and characterize these 12 isolates using molecular tools that included PCR based screening of *B. anthracis* specific prophages and chromosomal markers, sequencing of 16S rDNA and *pag* genes. We also employed canSNP analysis technique to delineate the clonal lineages for the set of *B. anthracis* isolates.

## Results and discussion

### 16S rDNA sequence analysis

*B. anthracis* genome is characterized by the presence of multiple rRNA operons [5]. Two specific rDNA positions, viz.*,* 1139 and 1148 with nucleotide mismatches among the eleven 16S rRNA operons have been reported earlier for the species identification [5, 6]. Base identification by visual inspection of electropherograms revealed dual peaks representing R (A or G) for the former and W (T or A) for the latter at these nucleotide mismatches (Table 1) [6]. These dual peaks specific to *B. anthracis* has been used for their identification when the consensus 16S rDNA gene sequence could not be used for differentiation of the closely related *B. cereus* sensu *lato strains* i.e., *B. cereus*, *B. anthracis* and *B. thuringiensis* with high level (> 99%) of sequence similarity [5]. The W at position 1148 (1146 in Sacchi et al. [5]) initially found to be specific for *B. anthracis* and grouping them in 16S rDNA type 6 [5]. The same position was found among *B. cereus* and *B. thuringiensis* strains, whereas the dual peak R at 1139 was found only in *B. anthracis* strains making this position more reliable for *B. anthracis* identification [6]. All the 12 *B. anthracis* strains characterized in this study had identical 16S rDNA nucleotide sequences with A at 1146 position (Table 1) indicative of 16S type 7 shared by *B. anthracis*, *B. cereus* and *B. thuringiensis* strains [5, 6]. Alternatively, the isolates had the *B. anthracis* specific dual peaks showing mixed base pair R (G/A) at position 1139 with visual inspection (Additional file 1) while the automated basecaller software indicated a G at this position (Table 2) as reported earlier [6]. Conversely, analysis of consensus 16S rDNA sequences ranging from nucleotide positions 352 to 1007 bp is short of the significant number of nucleotides at both 5′ and 3′ regions that otherwise held multiple polymorphic sites, could not discriminate *B. anthracis* from *B. cereus* and *B. thuringiensis* [23]. We further extended our analysis and performed multiple sequence alignment (MSA) with an additional 167 consensus 16S rDNA sequences and 3 sequences with base identification by visual inspection of *B. cereus* sensu *lato* to identify the microheterogeneity in the 16S rDNA gene. This included 47 strains including *B. anthracis*, *B. cereus B. thuringiensis* and *B. mycoides* which were representative of eleven 16S types reported by Sacchi et al. [5] scheme. Also, 33 *Bacillus* strains (15 inclusivity and 18 exclusivity species) mentioned in the Association of Analytical Communities (AOAC) International panel were included in the analysis [24]. Additionally, 90 strains including *B. anthracis*, *B. cereus*, *B. thuringiensis* and, *B. mycoides* for which 16S rDNA sequences were available from the GenBank database were included in the MSA. A total of 104 variant sites (V) which included 13 SNP positions reported earlier [5], 27 new parsimony-informative sites (Pi) and 64 new singletons (S) were observed in the 1402 bp 16S rDNA sequence examined. According to the 13 SNP positions [5], 50 of the additional 170 strains included in the study exhibited 19 new 16S types not described earlier [5]. Also, it was observed that 28 *B. anthracis* strains and 13 *B. thuringiensis* strains from the GenBank database were of 16S types 13 and 7 respectively, otherwise seen exclusively among *B. cereus* strains reported earlier [5] (Additional file 2). The position 1139 (1137 in Sacchi et al. [5]) was uniformly identified with G in most of the *B. cereus* sensu *lato* strains, with few exceptions. The exceptions included the twelve isolates from the present study and three *B. anthracis* strains, Pasteur, A0248 and Sterne that had R (G/A) derived from dual peaks for the mixed base pair observed on visual inspection of the electropherograms [6] (Table 2). We speculate that the use of consensus 16S rDNA sequence from automated base caller software could have possibly limited the detection of mixed base pair R (G/A) at position 1139 [6].

**Table 1:** The 16S rDNA genotypes of *Bacillus anthracis* isolates elucidated using nucleotide positions reported by Sacchi et al. [5]

Nucleotide Positions^**a**^														
16S type	***Bacillus*** Strain	1 (77)	2 (90)	3 (92)	4 (182)	5 (189)	6 (192)	7 (20)	8 (208)	9 (1015)	10 (1036)	11 (1045)	12 (1146)	13 (1462)
**1**	*mycoides*	A	T	T	C	C	C	G	C	C	C	G	A	–
**2**	*cereus*	R^**b**^	Y	W	C^**c**^	A	T	T	G	A	T	A	A	A
**3**	*cereus*	G	C	A	Y	A	T	T	G	A	T	A	A	A
**4**	*thuringiensis*	G	C	A	C	A	T	T	G	A	T	A	A	–
**5**	*cereus*	G	C	A	C	A	C	T	G	A	T	A	A	–
**6**	*anthracis*	A	T	T	C	A	C	T	G	C	T	A	W	T
**7**	*anthracis*	A	T	T	C	A	C	T	G	C	T	A	A	T
**7**	*cereus*	A	T	T	C	A	C	T	G	C	T	A	A	T
**9**	*cereus*	A	T	T	C	A	C	T	G	A	T	A	A	T
**10**	*thuringiensis*	A	T	T	C	A	Y	T	G	A	T	A	A	T
**12**	*cereus*	A	T	T	Y	A	T	T	G	A	T	A	A	T
**13**	*cereus*	A	T	T	C	A	C	T	G	C	T	A	T	T
**7**	***anthracis*DFR.BHE** **1–12**	A	T	T	C	A	C	T	G	C	T	A	A	T

^**a**^16S rDNA positions. Numbers in paranthesis refers to polymorphic positions in the 16S rDNA gene and are numbered from 1 to 13. ^**b**^**R** refers to a purine (A or G) at that position; Y refers to a pyrimidine (C or T) at that position; and W refers to an A or T at that position. ^**c**^A, C, G, and T refer to the four deoxynucleotides that DNA comprises. DFR.BHE refers strains from Bheemana Bedu village of Gundlupet region of Karnataka, India maintained in Defence Food Research Laboratory, Mysore, India repository

**Table 2:** The *Bacillus anthracis* 16S rDNA sequence results reported by Hakovirta et al. [6] using basecaller software and from visual inspection of electropherogram at position 1139

Species	No. of strains	No. of strains with result at nucleotide position 1139			
		Basecaller		Visual inspection	
		G	N	G	R (G/A)
*B. anthracis* isolates (this study)	12	12	0	0	12
*B. anthracis* strains (Hakovirta et al., 2016 [6] and this study)	3	3	0	0	3

Alternatively, when we considered the 27 new Pi sites (positions 14 to 40) along with the 13 previously identified SNP positions for characterizing the population, 28 new 16S types could be identified among the 182 strains (Additional file 2). However, even with the inclusion of 27 additional Pi sites/ SNPs, all the 47 *B. cereus* sensu *lato* strains from Sacchi et al. [5] scheme, 51 strains from GenBank population and the twelve isolates in the present study held on to eleven 16S types reported earlier [5]. About, 19 *B. anthracis* strains and 10 *B. cereus* strains were distributed in eight 16S types each viz.*,* 16S types A, C, G, H, I, M, R, T and 16S types J, L, N, U, V, W, X and Y respectively (Additional file 2). Five *B. thuringiensis* strains were distributed in four 16S types B, D, Z and AA while 5 *B. mycoides* strains in two 16S types Q and BB (Additional file 2). Interestingly, 8 *B. cereus* strains and 19 *B. thuringiensis* strains shared four 16S types E, F, O and P, whereas, 4 *B. cereus* strains and 2 *B. anthracis* strains shared two common 16S types, K and S respectively (Additional file 2). Inclusion of additional *B. cereus* sensu *lato* strains revealed newer polymorphic nucleotide positions resulting in new 16S rDNA types. Even though 16S rDNA types of *B. anthracis* strains could solely be identified (16S types A, C, G, H, I, M, R and T), few of them clustered along with *B. cereus* (16S types E, F, O and P) also. This could result in ambiguous identification of any *B. cereus* sensu *lato* strains clustering in 16S rDNA types with multiple species.

**Fig. 1:**
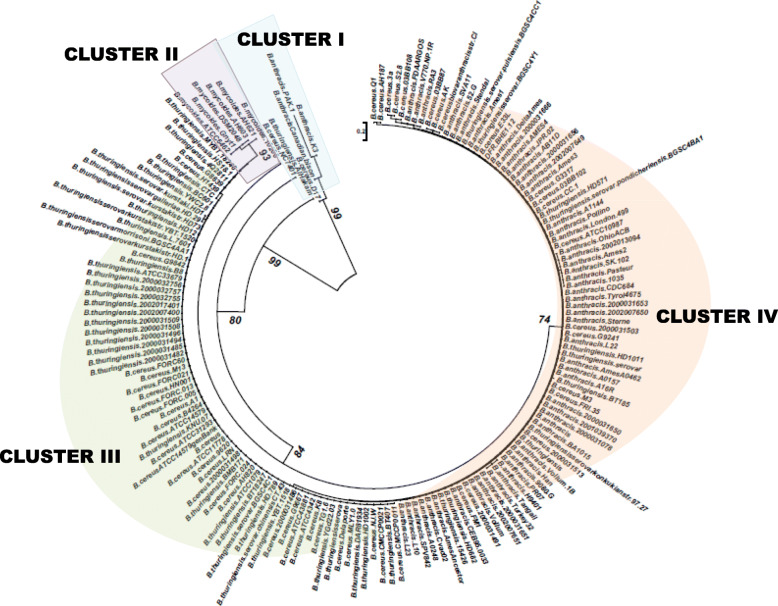
Phylogenetic relationship of *B. cereus* sensu *lato* based on 16S rDNA gene analyses. Neighbour joining tree representing *B. cereus* sensu *lato* phylogeny based on 16S rDNA gene. The regions shaded in color blue, purple, green and orange represent Cluster I, II, III and IV respectively. The tree is drawn to scale with branch lengths measured in the number of substitution per site. Numbers above branches are support values from 1000 bootstrap replicates. Evolutionary analyses were conducted using Neighbour Joining method in Mega X

Also, the Neighbor joining tree constructed with bootstrap values calculated from 1000 replications grouped the entire *Bacillus* population in the present study into four major clusters with an average pairwise distance of 0.054–0.182 (Fig. 1). The polymorphisms at nucleotide positions 19 to 35 resulted in Cluster I, an out-group with *B. anthracis* strains PAK.1, Canadian Bison, K3 and *B. cereus* strain D17 with 16S types A, G, M, and N respectively. This cluster also included *B. thurnigiensis* strain Al-Hakam and *B. cereus* strain NC7401 for which position 13 [5] could not be elucidated due to their shorter 16S rDNA sequences available in the GenBank. The Cluster II mainly consisted of *B. mycoides* strains and comprised of two new 16S types Q and BB along with 16S type 1 (*B. mycoides* ATCC 10206) identified earlier [5]. Cluster III comprised majorly *B. cereus* and *B. thuringiensis* strains representing the 8 new 16S types of *B. cereus*, 4 new 16S types of *B. thuringiensis* and two new 16S types shared among the two species. Cluster IV consisted of *B. anthracis* strains along with *B. cereus* and *B. thuringiensis* strains that shared *B. anthracis* plasmids and few other strains of these species. This cluster represented 8 new 16S types of *B. anthracis* and 2 new 16S types shared among *B. cereus* and *B. anthracis*. The twelve isolates in the present study also grouped in this cluster along with other *B. anthracis* strains with 16S type 7 and exhibited 100% similarity in the MSA with no gaps or mismatches in the entire 16S rDNA gene analyzed.

### Screening of *B. anthracis* specific loci and prophages

*B. anthracis* strains are routinely identified based on the presence of plasmid-borne virulence marker genes (*pag* and *cap*) [25]. However, the existence of either or both of these plasmids or their homologs in certain *B. cereus* sensu *lato* strains poses challenges in the confirmatory identification of *B. anthracis*. Assays targeting polymorphism present in chromosome borne housekeeping genes such as *rpoB* [26, 27], *gyrA* [28], *gyrB* [29], *sspE* [30] and *sap* [31] have been used to distinguish *B. anthracis* from other species of *B. cereus senu stricto* group. Four putative lambdoid prophages (designated lambdaBa01, lambdaBa02, lambdaBa03 and lambdaBa04) have been reported to be integrated specifically in the 3% of 5.2 Mb *B. anthracis* chromosome [8]. They are conserved across more than 300 geographically and temporally divergent *B. anthracis* strains [32].

The presence of all the four prophages in the chromosome is a unique feature used for definitive discrimination of *B. anthracis* from certain *B. cereus* sensu stricto strains that contain some of these lambdoid prophages but with few sequence homologies with *B. anthracis* prophage genes [8]. *B. cereus* sensu *lato* strains such as *B. cereus* E33L and *B. weihenstephanensis* KBAB4 have been reported to contain *B. anthracis* lambda01 prophage in their chromosome [33] and hence can be determined as non-anthrax causing *Bacillus* species. Also the BLAST results of these four lambdoid prophages indicated their presence in all the *B. anthracis* isolates present in the NCBI database and no other *Bacillus* species were found to contain all of the prophages in their genome (data not shown). However, the *B. anthracis* isolates in the present study were confirmed for the presence of all the four prophage regions yielding expected amplicon sizes (221, 233, 189 and 157 bp) for lambdaBa01, lambdaBa02, lambdaBa03 and lambdaBa04 markers respectively (Additional file 3A).

Similarly, the presence of four unique “DNA signatures” A, C, D, and E (dhp 61.183, dhp 77.002, dhp 73.019, and dhp 73.017 respectively) within the *B. anthracis* genome is considered as a powerful tool to differentiate *B. anthracis* from non-anthrax causing *Bacillus* species [7]. In the present study, we found that the DNA of all the twelve *B. anthracis* isolates consistently amplified all the four *B. anthracis* specific gene markers, dhp 61.183, dhp 77.002, dhp 73.019 and dhp 73.017 with the predicted amplicon size (163 bp, 133 bp, 196 bp, and 241 bp respectively) (Additional file 3B) with no variations within the isolates. These findings corroborated the identification of the *B. anthracis* isolates in the present study.

### Protective antigen gene *(pag)* sequence analysis and genotyping

Protective antigen (PA), the binding component of anthrax toxin is encoded by *pag* gene, located on pXO1 plasmid of *B. anthracis* [34]. *B. anthracis* strains have been classified into six PA genotypes based on the polymorphism in 7 nucleotide positions observed in the 2294 bp *pag* gene (GI: NC_001496.1) from 26 diverse *B. anthracis* strains [35]. These seven nucleotide positions included four synonymous and three missense mutations resulting in four different phenotypes. Therefore, to characterize the PA genotype and phenotype, the *pag* amplicons from all the 12 isolates were sequenced and analyzed. The PA genotyping helped in elucidating any variation in the gene resulting from gene alteration or gene engineering and hence, aided in understanding the evolution of our *B. anthracis* isolates [36]. Also, the *pag* sequences from recently evolved *B. anthracis* strains and *B. anthracis* like strains, which were not included in the Price et al. [35] scheme, were analyzed to evaluate the phylogenetic relationship of our twelve isolates with the global *B. anthracis* strains in the present scenario. The *pag* sequences of the twelve isolates, 26 *B. anthracis* strains from the earlier scheme [35], 15 *B. anthracis* strains from the inclusivity panel by AOAC [24], 5 *B. cereus* strains with pXO1 like plasmids [37] and 14 *B. anthracis* strains from the GenBank were analyzed together for their phylogenetic comparisons. *B. anthracis* isolates of the present study were found to be of *pag* genotype I and FPA phenotype (Table 3) representing the Sterne-Ames diversity group as described previously [35]. This *pag* genotype I have been reported previously in 135 *B. anthracis* isolates from bioterrorism associated anthrax outbreak in the United States during 2001 [36], human anthrax cases from Hong Kong [38] and Lianyungang regions [39], and cattle related anthrax outbreak from Central Java and Yogyakarta regions of Indonesia [40]. No new *pag* genotypes other than that reported by Price et al. [35] scheme were identified among *B. anthracis* even on including additional strains from recent outbreaks.

**Table 3:** Protective antigen genotype and phenotype identified among *Bacillus anthracis* strains according to Price et al. [35] typing scheme

PA Genotype	Diversity Groups	Mutations^**a**^						
		1	2	3	4	5	6	7
I	Sterne- Ames	C	G	T (F)	C (P)	C (A)	T	A
II	Sterne- Ames	C	G	T (F)	C (P)	C (A)	C	A
III	Southern Africa	T	G	T (F)	C (P)	C (A)	T	A
IV	Southern Africa	T	A	T (F)	C (P)	C (A)	T	A
V	Southern Africa	T	G	T (F)	C (P)	T (V)	T	A
V	Kruger	T	G	T (F)	C (P)	T (V)	T	A
V	WNA	T	G	T (F)	C (P)	T (V)	T	A
V	Vollum	T	G	T (F)	C (P)	T (V)	T	A
VI	Vollum	T	G	T (F)	T (S)	T (V)	T	A
**I**	**DFRL.BHE 1–12** **(Sterne-Ames)**	C	G	T (F)	C (P)	C (A)	T	A

^**a**^According to Price et al. [35]

However, the sequence clustering on the inclusion of 34 additional *pag* sequences along with the *pag* sequence data of 12 isolates revealed two additional polymorphic nucleotide positions among the 5 *B. cereus* strains with pXO1 like plasmids causing missense mutations at nucleotide positions 196 bp and 869 bp of the 2294-bp *pag* sequence from Sterne strain (GI: NC_001496.1) (Table 4). No variations among *B. anthracis* strains were found at these two additional positions of differences. The *pag* is divided into 4 domains where each domain plays a critical role in toxin action [41]. The transition mutation (T to C) at the nucleotide position 196 resulted in a serine to proline change near the amino-terminal fragment (PA_20_) of PA domain 1 (amino acids (aa) 1 to 258) that is cleaved due to furin protease before the binding of the PA_63_ monomer to the host cell receptor [41]. Alternatively, the isoleucine to serine change in aa 289, a part of PA domain 2 (aa 259 to 487) brought about by the transversion mutation (T to G) at 869 bp polymorphic site, could possibly play a role in altering the binding of PA_63_ monomer and needs further elucidation [42].

**Table 4:** Additional mutations identified in the *pag* gene sequence of *Bacillus cereus* strains used in the present study

Nucleotide position^**a**^ of polymorphism	Base change	Amino acid change
**196**	**T↔C**	**S↔P**
**869**	**T↔G**	**I↔S**

^**a**^Nucelotide positions based on the 2294 bp *pag* sequence from Sterne strain (GI: NC_001496.1)

**Fig. 2:**
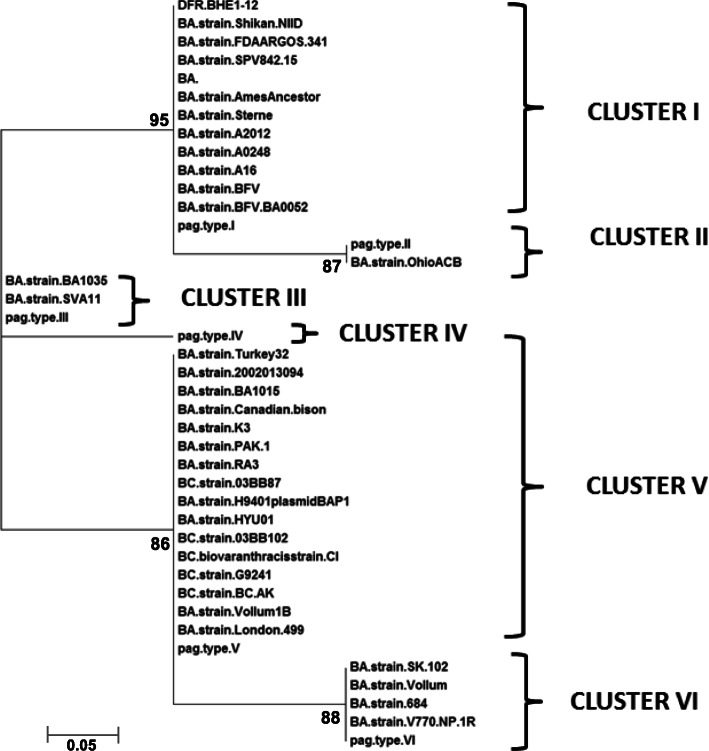
Phylogenetic analysis of *Bacillus anthracis* DFR.BHE strains 1–12 based on protective antigen (*pag*) gene. Unrooted, maximum likelihood phylogenetic tree constructed on the basis of *pag* gene sequences of *B. anthracis (BA)* strains and *B. cereus (BC)* strains with pXO1 like plasmids. The six PA genotypes are represented with six clusters (I-VI)

The Neighbour joining tree was constructed based on nucleotide variations at 7 polymorphic sites studied with bootstrap values calculated from 1000 replications grouped the entire *B. anthracis* group of strains population in the present study into six major clusters with an average pairwise distance of 0.168–0.973 (Table 5). Clusters I, II, III, IV, V and VI were represented by *pag* types I, II, III, IV, V and VI respectively (Fig. 2). The twelve *B. anthracis* isolates from the present study grouped with *pag* type I along with *B. anthracis* strain Ames Ancestor and Sterne. The five *B. cereus* strains with *pag* gene analyzed in the present study were grouped in Cluster V of *pag* type V excluding the two additional positions of mutations in consideration.

**Table 5:** Estimates of evolutionary divergence over sequence pairs between groups for *pag* gene. The number of base substitutions per site from averaging over all sequence pairs between groups are shown. Standard error estimate(s) are shown above the diagonal. Analyses were conducted using the Kimura 2-parameter model. The analysis involved 61 nucleotide sequences. All positions containing gaps and missing data were eliminated. Evolutionary analyses were conducted in MEGA X [43]

CLUSTER NAMES	IV	II	I	VI	V	III
**IV**		**0.390**	**0.343**	**0.401**	**0.346**	**0.237**
**II**	**0.973**		**0.228**	**0.395**	**0.394**	**0.340**
**I**	**0.424**	**0.168**		**0.402**	**0.341**	**0.244**
**VI**	**0.973**	**0.424**	**0.973**		**0.236**	**0.331**
**V**	**0.424**	**0.973**	**0.424**	**0.168**		**0.230**
**III**	**0.168**	**0.424**	**0.168**	**0.424**	**0.168**

### canSNP genotyping

**Fig. 3:**
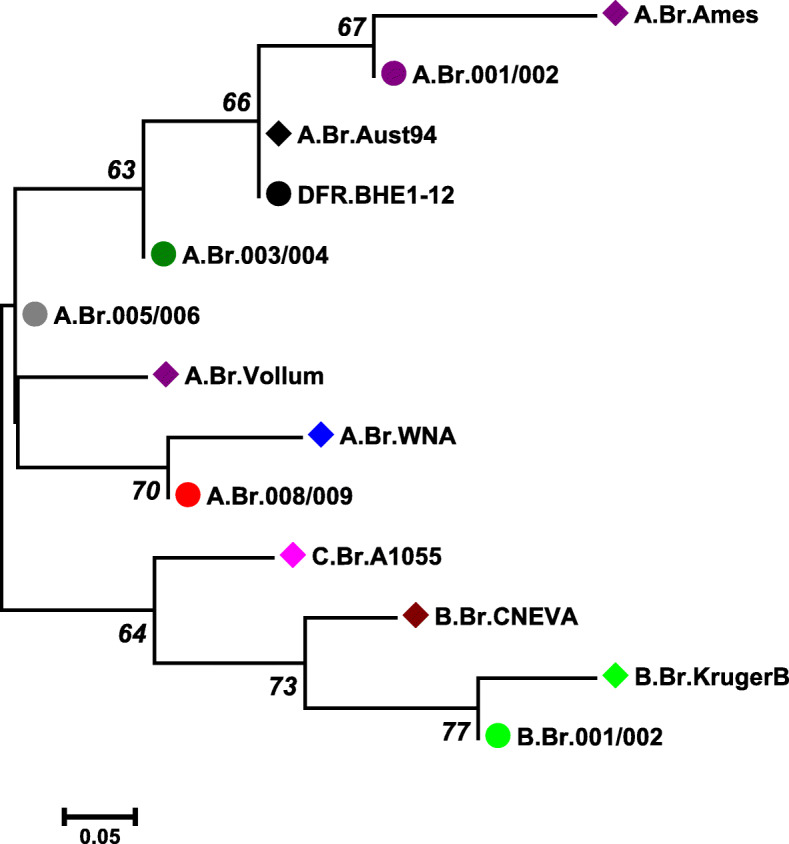
Phylogeny of 12 canonical single nucleotide polymorphisms (canSNP) sub-lineages and the twelve *Bacillus anthracis* strains (DFR.BHE 1–12). Terminal sub groups representing specific lineages are shown as diamonds, and intervening nodes representing collapsed branches appear as circles. All 13 canonical SNPs were concatenated into a single nucleotide sequence for each subgroup and strain. The trees were generated from an alignment (gap open cost, 15; gap extension cost, 6.66; end gap cost, free) using a maximum likelihood method with 1000 bootstrap replicates (MEGA X)

SNPs have played a major role as a genetic marker for detecting and subtyping bacterial pathogens [44]. A small number of SNPs have been used to define genetic groups efficiently even among largely monophyletic clade like *B. anthracis*. The phylogenetic positions of *B. anthracis* isolates were evaluated using the 13 canSNPs scheme as proposed earlier (Table 6) [9]. All the *B. anthracis* isolates from the study were assigned to sub-lineage A.Br.Aust 94, a part of major clonal lineage A of *B. anthracis* (Fig. 3). Members of this sub-lineage have been identified throughout the five continents viz., Australia, Africa (South Africa, Namibia, Mozambique), America (USA), Europe (Germany, Great Britain, The Netherlands) and Asia (China, Turkey, Georgia, Thailand, India) [9]. The origin of this lineage has been reported in Asia and/or Middle East such as India, China and Turkey [9] and had possibly spread through the ancient trade route known as the Silk Road that extends from Europe, the Middle East and portions of Asia [45]. The A.Br.Asut94 sub-lineage is also associated with anthrax from humans and non-bovines such as zebra, swine [45], sheep and dog [46].

**Table 6:** Canonical single nucleotide polymorphisms (canSNPs) used for delineating *Bacillus anthracis* sub-lineages and corresponding nucleotides identified among the isolates from the present study

***B. anthracis*** sublineages	13 canonical SNP sites												
	A.Br.001	A.Br.002	A.Br.003	A.Br.004	A.Br.006	A.Br.007	A.Br.008	A.Br.009	B.Br.001	B.Br.002	B.Br.002	B.Br.002	A/ B.Br.001
**C.Br.A1055**	T	G	A	T	C	T	T	A	T	G	G	T	G
**B.Br.KrugerB**	T	G	A	T	C	T	T	A	C	T	A	T	A
**B.Br.001/002**	T	G	A	T	C	T	T	A	T	T	A	T	A
**B.Br.CNEVA**	T	G	A	T	C	T	T	A	T	G	A	C	A
**A.Br.Ames**	C	A	G	C	A	T	T	A	T	G	C	T	A
**A.Br.001/002**	T	A	G	C	A	T	T	A	T	G	G	T	A
**A.Br.Aust94**	T	G	G	C	A	T	T	A	T	G	G	T	A
**A.Br.003/004**	T	G	A	C	A	T	T	A	T	G	G	T	A
**A.Br.Vollum**	T	G	A	T	A	C	T	A	T	G	G	T	A
**A.Br.005/006**	T	G	A	T	A	T	T	A	T	G	G	T	A
**A.Br.008/009**	T	G	A	T	A	T	G	A	T	G	G	T	A
**A.Br.WNA**	T	G	A	T	A	T	G	G	T	G	G	T	A
**DFRL.BHE 1–12**^**a**^ **(A.Br.Aust94)**	T	G	G	C	A	T	T	A	T	G	G	T	A

^**a**^DFRL.BHE 1–12 refers *B. anthracis* DFRL.BHE 1–12 strains from this study

In contrast to other regions of the world that are extensively sampled [47–50], the Indian sub-continent is still poorly studied [51]. The southern states of the sub-continent are endemic to the disease due to unprotected livestock population and also attributable to warm humid climate, alkaline calcareous soil favoring survival and germination of anthrax spores [52]. The high density of livestock population favors the transmission of disease between more susceptible animals and less susceptible human beings. Multiple animal or human cutaneous anthrax outbreaks from India at Vellore [53], Chittoor [20], Ramanathapuram [54], and Visakhapatnam [21] have been reported earlier, but without sub-lineage analysis of the *B. anthracis* isolates. However, the 10 Indian *B. anthracis* isolates that have been delineated as A.Br.Aust94 sub-lineage by canSNP analysis could not be attributed to any of these outbreaks [9]. Consequently, the present study attributes A.Br.Aust94 lineage of *B. anthracis* to the anthrax outbreak among cattle in Karnataka state and also reiterates the prevalence of this lineage in India as reported earlier [9].

## Conclusions

In summary, we can conclude that all the twelve *B. anthracis* isolates shared similar 16S type 7 and *pag* type I. Our findings also revealed that all the isolates examined belonged to clonal lineage A (worldwide lineage), specifically the sub-lineage A.Br.Aust94 similar to previously characterized strains from the Indian sub-continent. Collectively, the genotyping findings evidenced the fact that the anthrax outbreak cases in Gundlupet region might have originated from a common source of infection.

## Methods

### Bacterial strains, growth conditions and DNA extraction

The bacterial cultures used in the present study isolated and identified as *B. anthracis* earlier [22] are listed in Additional file 4. The *Bacillus* strains were maintained in Brain Heart Infusion (BHI) broth and incubated at 37 °C with shaking. All the media were procured from Hi-Media, India. Antibiotics were procured from Sigma-Aldrich (India). The *B. cereus* ATCC 14579 strain and *B. anthracis* BA10 strain from Defence Food Research Laboratory (DFRL), Mysore, India repository were used as the negative and positive controls respectively, for all the PCR experiments. Total genomic DNA (Additional file 4) was extracted from every bacterial culture using Gen Elute Bacterial Genomic DNA Kit (Sigma, India) following the manufacturer’s instructions. The DNA samples thus obtained were diluted to a final concentration of 100 ng/μl for PCR experiments.

### 16S rDNA sequencing

The 16S rDNA gene sequencing was performed for the 12 *B. anthracis* isolates using universal bacterial primers, 16S-27F and 16S-1488R (Additional file 5) [55]. All PCR reactions were performed in Bio-Rad S1000 thermal cycler (Bio-Rad, USA) wherein the samples were maintained at 94 °C for 4 min for initial denaturation followed by 30 cycles of denaturation at 94 °C for 30 s, annealing at 56 °C for 45 s, extension at 72 °C for 1 min and a final extension at 72 °C for 8 min. PCR products were then gel purified using Gen Elute gel extraction Kit (Sigma, India) and sequenced in both forward and reverse directions with 16S-27F and 16S-1488R primers using BigDye Terminator v3.1 Cycle sequencing kit (Applied Biosystems, USA). Nucleotide sequences were obtained using Sequencing Analysis Software v5.4 in ABI 3500 Genetic Analyzer (Applied Biosystems, USA). These sequence data have been submitted to the GenBank databases under accession numbers MN421996-MN422007. The 16S rDNA nucleotide sequences from additional 170 strains of *B. cereus* sensu *lato* (Additional file 6) were included in the present study for phylogenetic comparisons of the 12 *B. anthracis* isolates of the present study. This collection included 90 *B. cereus* sensu *lato* strains from GenBank (28 *B. cereus* strains, 28 *B. anthracis* strains, 30 *B. thuringiensis* strains, and 4 *B. mycoides* strains); 33 *Bacillus* strains (15 inclusivity and 18 exclusivity strains) as per Association of Analytical Communities (AOAC) International panel [24] and 47 *Bacillus* strains studied from Sacchi et al. [5] scheme. A total of 182 (170 from the GenBank and 12 of the isolates under study) 16S rDNA gene sequences were analyzed in this study. Phylogenetic analysis was performed using MEGA software, version X [43] to form a Neighbor-Joining tree of all the 182 *B. cereus* sensu *lato* 16S rDNA gene sequences thus compiled. When applied, bootstrap analysis was computed with 1000 replicates.

### Detection for the presence of *B. anthracis* prophages and *B. anthracis* specific loci

The presence of *B. anthracis* specific prophages (lambdaBa01, lambdaBa02, lambdaBa03, lambdaBa04) were examined as described earlier with slight modifications for *B. anthracis* strains from India (DFR.BHE 1–12) [22]. Briefly, all the PCR reactions were carried out in Bio-Rad S1000 thermal cycler (Bio-Rad, USA) under the following conditions: 94 °C initial denaturation for 4 min, and 72 °C final elongation for 8 min, with 30 cycles of 94 °C for 1 min, 52 °C for 45 s, and 72 °C for 1 min. Additionally, the four *B. anthracis* specific loci (A, C, D and E) were simultaneously detected in the genomes of all *B. anthracis* isolates as described earlier [7]. The PCR amplicons were electrophoresed in 2% agarose gel, stained with ethidium bromide and visualized under UV transillumination (G-box, Syngene, India). Primer sequences along with their respective amplicon sizes are listed in Additional file 5.

### Protective Antigen (PA) sequencing

**Fig. 4:**
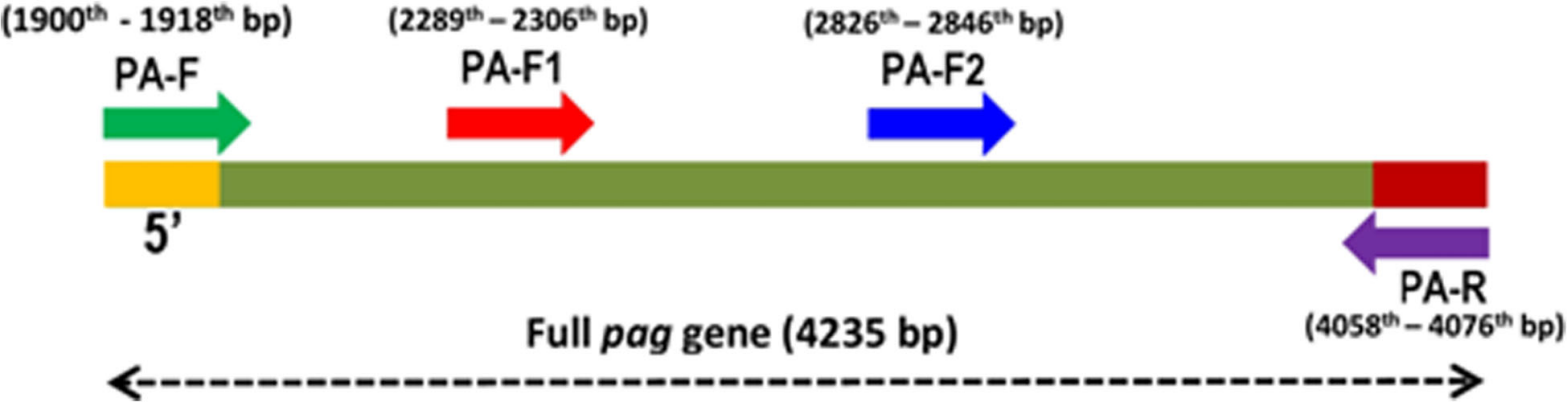
Strategy for *pag* cloning and sequencing. The locations of primers (this study) used for PA sequencing are shown with arrows on the full *pag* gene (4235 bp) of *B. anthracis.* Arrow color: Green indicates PA-F (1900–1918 bp); Red indicates PA-F1 (2289–2306 bp); Blue indicates PA-F2 (2826–2846 bp), and Purple indicates PA-R (4058–4076 bp)

The 2158 bp region of the protective antigen gene (*pag*) (GenBank accession No. M22589) was amplified by PCR using PA-F and PA-R primers (this study). The primers were designed in the present study using Gene runner ver. 5.0 (Additional file 5) to amplify *pag* from all the twelve *B. anthracis* isolates along with the DNA of control *B. anthracis* BA10. The PCR reactions were maintained at 94 °C initial denaturation for 4 min and 72 °C final elongation for 8 min, followed by 30 cycles of 94 °C for 1 min, 56 °C for 1 min and 72 °C for 2 min 30 s. PCR products were then gel purified using Gen Elute gel extraction Kit (Sigma, India). The purified amplicons (PA-F and PA-R) were then cloned and sequenced using the custom synthesized primers (Sigma, India) from the *pag* sequence (GenBank accession No. M22589) following the strategy as depicted in Fig. 4. Briefly, the purified products were cloned in pTZ57R/T vector using an InsTA clone PCR cloning kit (Thermo Scientific, India) according to manufacturer’s instructions and the recombinant plasmids were extracted using Gen Elute Plasmid Miniprep Kit (Sigma, India). Bidirectional sequencing was then performed on the cloned *pag* amplicons using M13 F and M13 R primers (Additional file 5). Additionally, the primer walking strategy was followed using the primers PA-F1 and PA-F2 to sequence the intermediate regions of this cloned fragment starting from 2289th and 2826th bp positions respectively (based on GenBank accession No. M22589). The sequencing reads thus obtained using the four sequencing primers were then spliced to omit the overlapping nucleotide regions and form a single consensus sequence. In silico analysis was then carried out to screen the seven significant nucleotide positions according to the Price et al. [35] scheme for *pag* genotyping. These sequence data have been submitted to the GenBank databases under accession numbers MN447720-MN447731.

A collection of additional *pag* sequences from 29 *B. anthracis* strains and 5 *B. cereus* strains for which *pag* gene sequence data was available in NCBI database and were taken for the phylogenetic comparison along with the 12 *B. anthracis* DFR.BHE strains 1–12 in the present study. A total of these 46 *pag* gene sequences were determined and in silico screening for seven significant positions of mutations [35] was carried out. The seven SNPs thus obtained were concatenated into a single consensus nucleotide sequence for each isolate and further used for the maximum likelihood tree construction from an alignment with 1000 replicates using MEGA software, version X [43].

### CanSNP genotyping

All the twelve *B. anthracis* DFR.BHE strains 1–12 were genotyped as described [9] using the 13 canonical (can) SNP loci (Additional file 5). PCR amplifications were performed under following conditions: 4 min at 94 °C initial denaturation and 72 °C final elongation for 8 min with 30 cycles of 94 °C for 30 s, 56 °C for 30 s and 72 °C for 30 s. The amplicons thus obtained were cloned in pTZ57R/T vector as described previously in section 2.4 and sequenced subsequently using M13F and M13R primers (Additional file 5). The nucleotide sequences thus obtained were further screened in silico for the 13 significant nucleotide positions. The 13 identified canSNPs were concatenated into a single consensus nucleotide sequence for each isolate and were used for the maximum likelihood tree construction from an alignment (gap open cost, 15; gap extension cost, 6.66; end gap cost, free) with 1000 replicates using MEGA software, version X [43].

## Supplementary information

**Additional file 1. **Representative electropherograms of the *Bacillus anthracis* 16S rDNA gene sequences. The electropherogram of 16S rDNA reverse complement read of *B. anthracis* DFR.BHE 3 strain. Arrows indicate (a) single peak corresponding to position 1148 indicating A (T in reverse read here) (b) dual peak at position 1139 indicating mixed base pair G/A [C/T)in reverse read here] described by Hakovirta et al. 2016 [6].

**Additional file 2. **Additional nucleotide positions of mutations in the *Bacillus cereus* sensu *lato* 16S rDNA gene found in the present study. Numbers in paranthesis refers to polymorphic positions in the 16S rDNA gene and are numbered from 1 to 40. The 28 new 16S types are highlighted with colored boxes. The new genotypes of *B. anthracis*, *B. cereus*, *B. thuringiensis* and *B. mycoides* are boxed in green, orange, blue and yellow color respectively. The 16S types shared by both *B. cereus* and *B. thuringiensis* are boxed in purple color. The genotypes shared by both *B. cereus* and *B. anthracis* are boxed in red color.

**Additional file 3. **PCR for the screening of *Bacillus anthracis* specific prophages and loci in the 12 *B. anthracis* DFR.BHE strains 1–12. A. PCR with *B. anthracis* specific prophage genes, (a).lambda01, (b). lambda02, (c). lambda03 and (d). lambda04 and B. PCR with *B. anthracis* specific loci (a) dhp 61.183 (loci A), (b). dhp 77.002 (loci C), (c). dhp 73.019 (loci D), and (d). dhp 73.017 (loci E). Lane 1–12, *B. anthracis* DFRL.BHE strains 1–12 along with positive (Lane P: *B. anthracis* BA10) and negative (Lane N: *B. cereus* ATCC 14579) controls. Lane M: 100 bp molecular marker.

**Additional file 4.** List of bacterial cultures used in the present study.

**Additional file 5.** List of primers and their amplicons size used in the present study.

**Additional file 6. **List of strains and their accession numbers used for multiple sequence alignment of 16S rDNA gene and *pag* gene in the present study.

## Data Availability

All data generated or analyzed during this study are included in this published article and its supplementary information.

## Abbreviations

canSNP: Canonical Single Nucleotide Polymorphism
MR: Mortality Rate
BHI: Brain Heart Infusion
PCR: Polymersae Chain Reaction
SPADA: Stakeholder Panel on Agent Detection Assays
AOAC: Association of Analytical Communities
*pag*: Protective Antigen Gene
V: Variant site
Pi: Parsimonious informative site
S: Singleton site
BLAST: Basic Local Alignment Search Tool
NCBI: National Center for Biotechnology Information
PA: Protective antigen
NGS: Next Generation sequencing
NADRES: National Animal Disease Referral Expert System
NCDC: National Centre for Disease Control
ICAR-NIVEDI: Indian Council of Agricultural Research - National Institute of Veterinary Epidemiology and Disease Informatics
CDC: Centers for Disease Control and Prevention
GI: Gastrointestinal
