# Supplementary material for: Molecular characterization of B. anthracis isolates from the anthrax outbreak among cattle in Karnataka, India
Source: BMC Microbiol. 2020 Jul 31;20:232. doi: 10.1186/s12866-020-01917-1 (PMC7394690; doi:10.1186/s12866-020-01917-1)

Additional File 1: Representative electropherograms of the *Bacillus anthracis* 16S rDNA gene sequences. The electropherogram of 16S rDNA reverse complement read of *B. anthracis* DFR.BHE 3 strain. Arrows indicate (a) single peak corresponding to position 1148 indicating A (T in reverse read here) (b) dual peak at position 1139 indicating mixed base pair G/A [C/T)in reverse read here] described by Hakovirta et al. 2016 [6].

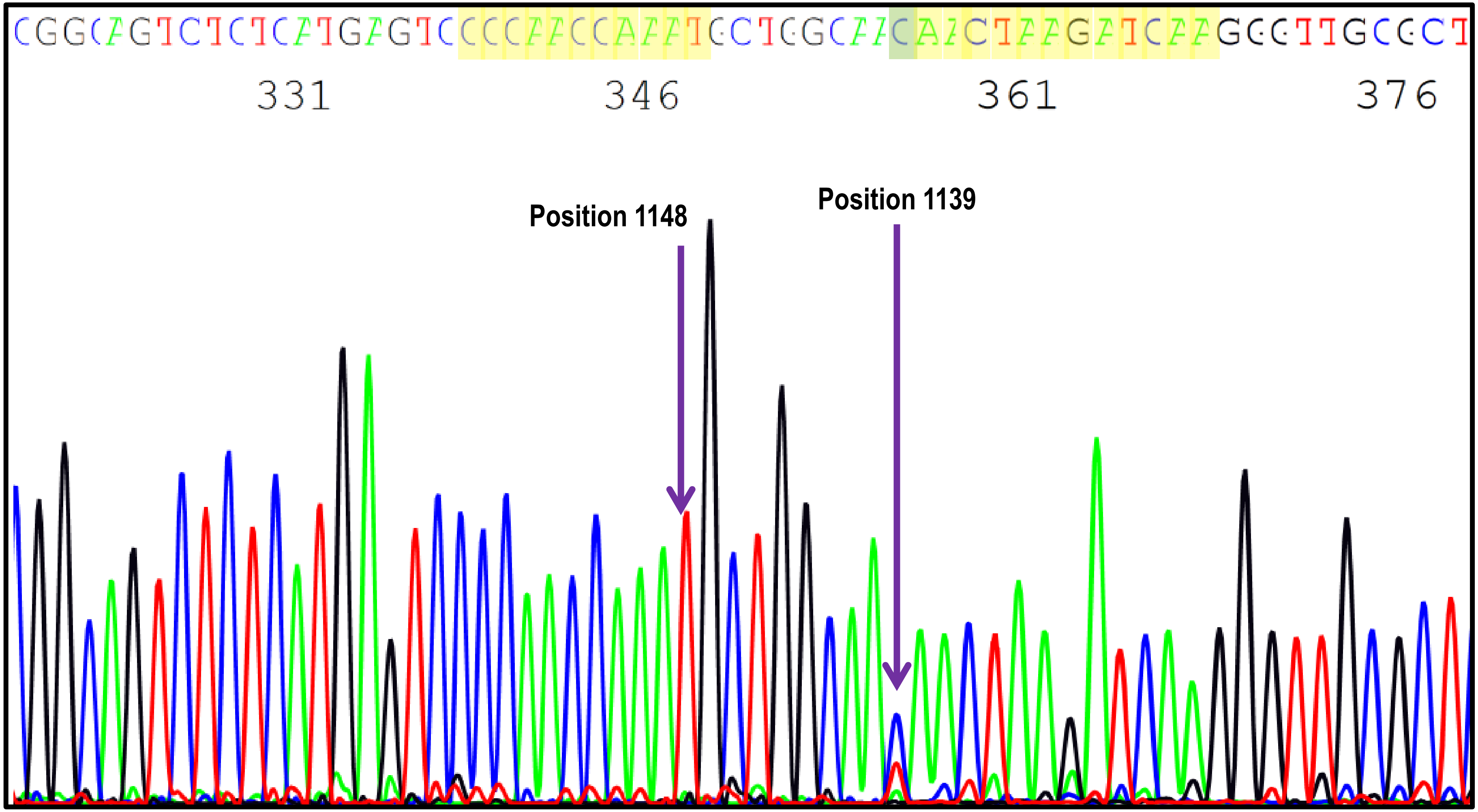

Supplement: Supplementary file 1 — Additional file 1. Representative electropherograms of the Bacillus anthracis 16S rDNA gene sequences. The electropherogram of 16S rDNA reverse complement read of B. anthracis DFR.BHE 3 strain. Arrows indicate (a) single peak corresponding to position 1148 indicating A (T in reverse read here) (b) dual peak at position 1139 indicating mixed base pair G/A [C/T)in reverse read here] described by Hakovirta et al. 2016 [6]. [file 12866_2020_1917_MOESM1_ESM.pdf]
