# Supplementary material for: Molecular characterization of B. anthracis isolates from the anthrax outbreak among cattle in Karnataka, India
Source: BMC Microbiol. 2020 Jul 31;20:232. doi: 10.1186/s12866-020-01917-1 (PMC7394690; doi:10.1186/s12866-020-01917-1)
Supplement: Supplementary file 2 — Additional file 2. Additional nucleotide positions of mutations in the Bacillus cereus sensu lato 16S rDNA gene found in the present study. Numbers in paranthesis refers to polymorphic positions in the 16S rDNA gene and are numbered from 1 to 40. The 28 new 16S types are highlighted with colored boxes. The new genotypes of B. anthracis, B. cereus, B. thuringiensis and B. mycoides are boxed in green, orange, blue and yellow color respectively. The 16S types shared by both B. cereus and B. thuringiensis are boxed in purple color. The genotypes shared by both B. cereus and B. anthracis are boxed in red color. [file 12866_2020_1917_MOESM2_ESM.docx]

**Additional File 2: Additional nucleotide positions of mutations in the *Bacillus cereus sensu lato* 16S rDNA gene found in the present study.**

**Note:** Numbers in paranthesis refers to polymorphic positions in the 16S rDNA gene and are numbered from 1 to 40. The 28 new 16S types are highlighted with colored boxes. The new genotypes of *B. anthracis*, *B. cereus*, *B. thuringiensis* and *B. mycoides* are boxed in green, orange, blue and yellow color respectively. The 16S types shared by both *B. cereus* and *B. thuringiensis* are boxed in purple color. The genotypes shared by both *B. cereus* and *B. anthracis* are boxed in red color.
