# Supplementary material for: Molecular characterization of B. anthracis isolates from the anthrax outbreak among cattle in Karnataka, India
Source: BMC Microbiol. 2020 Jul 31;20:232. doi: 10.1186/s12866-020-01917-1 (PMC7394690; doi:10.1186/s12866-020-01917-1)
Supplement: Supplementary file 3 — Additional file 3. PCR for the screening of Bacillus anthracis specific prophages and loci in the 12 B. anthracis DFR.BHE strains 1–12. A. PCR with B. anthracis specific prophage genes, (a).lambda01, (b). lambda02, (c). lambda03 and (d). lambda04 and B. PCR with B. anthracis specific loci (a) dhp 61.183 (loci A), (b). dhp 77.002 (loci C), (c). dhp 73.019 (loci D), and (d). dhp 73.017 (loci E). Lane 1–12, B. anthracis DFRL.BHE strains 1–12 along with positive (Lane P: B. anthracis BA10) and negative (Lane N: B. cereus ATCC 14579) controls. Lane M: 100 bp molecular marker. [file 12866_2020_1917_MOESM3_ESM.zip › Additional File 3 supplementary.pdf]

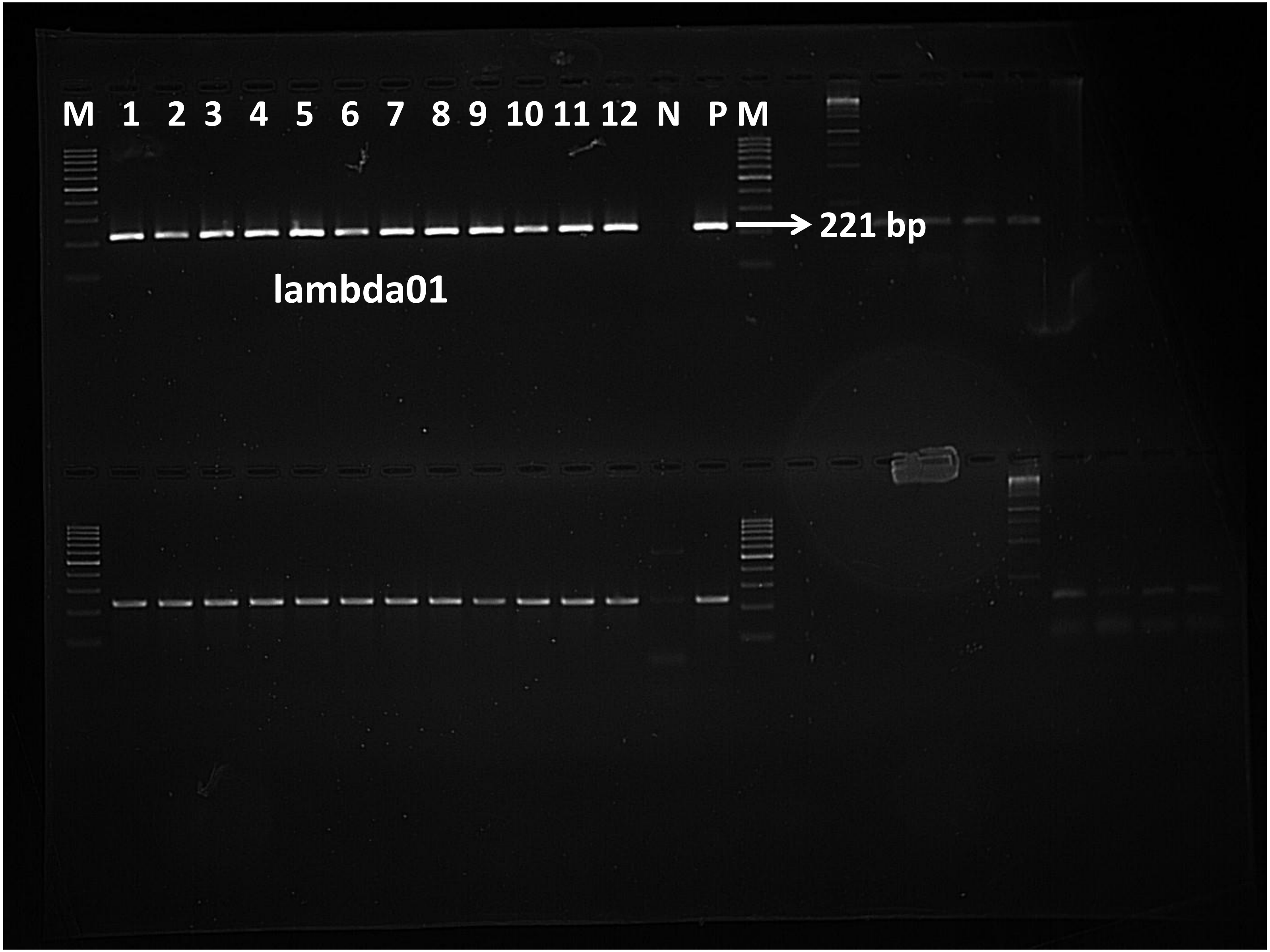

Additional File 3A. PCR for the screening of *Bacillus anthracis* specific prophages in the 12 *B. anthracis* DFR.BHE strains 1-12.
(a) lambda01
Lane 1-12, *B. anthracis* DFRL.BHE strains 1-12 along with positive (Lane P: *B. anthracis* BA10) and negative (Lane N: *B. cereus* ATCC 14579) controls. Lane M : 100 bp molecular marker.

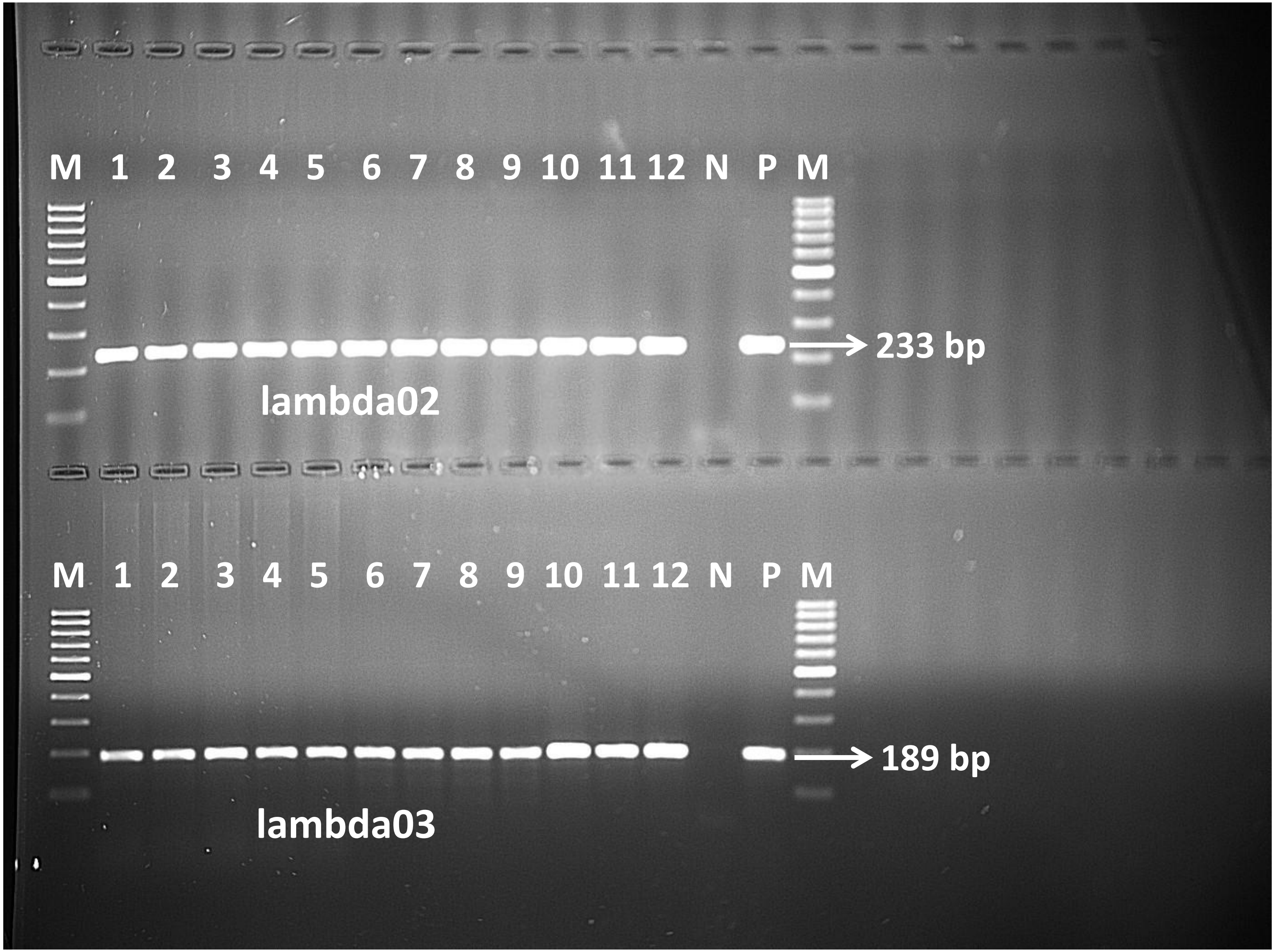

Additional File 3A. PCR for the screening of *Bacillus anthracis* specific prophages in the 12 *B. anthracis* DFR.BHE strains 1-12.
(b) lambda02 (c) lambda03

Lane 1-12, *B. anthracis* DFRL.BHE strains 1-12 along with positive (Lane P: *B. anthracis* BA10) and negative (Lane N: *B. cereus* ATCC 14579) controls. Lane M : 100 bp molecular marker.

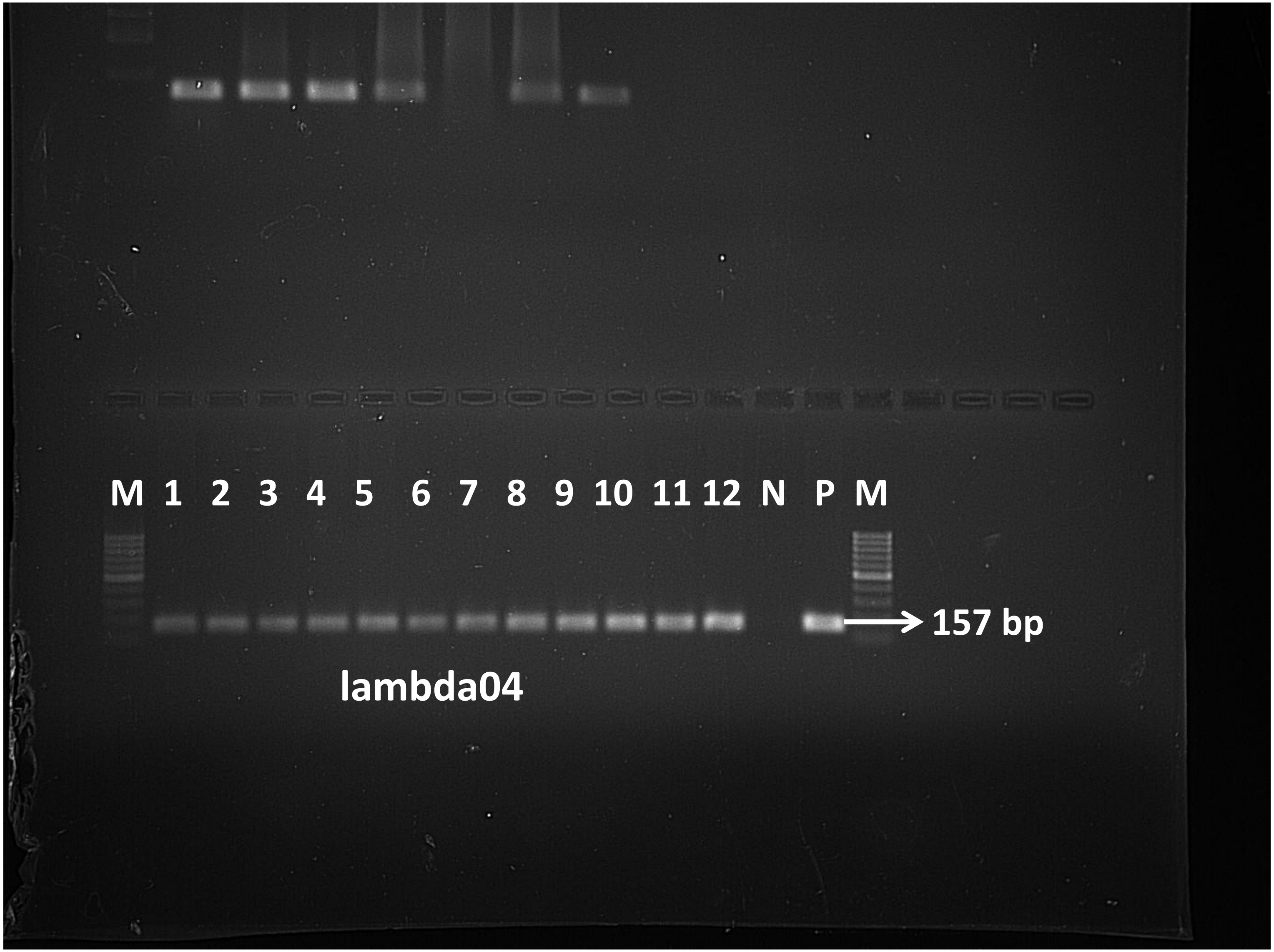

Additional File 3A. PCR for the screening of *Bacillus anthracis* specific prophages in the 12 *B. anthracis* DFR.BHE strains 1-12.
(d) lambda04
Lane 1-12, *B. anthracis* DFRL.BHE strains 1-12 along with positive (Lane P: *B. anthracis* BA10) and negative (Lane N: *B. cereus* ATCC 14579) controls. Lane M : 100 bp molecular marker.

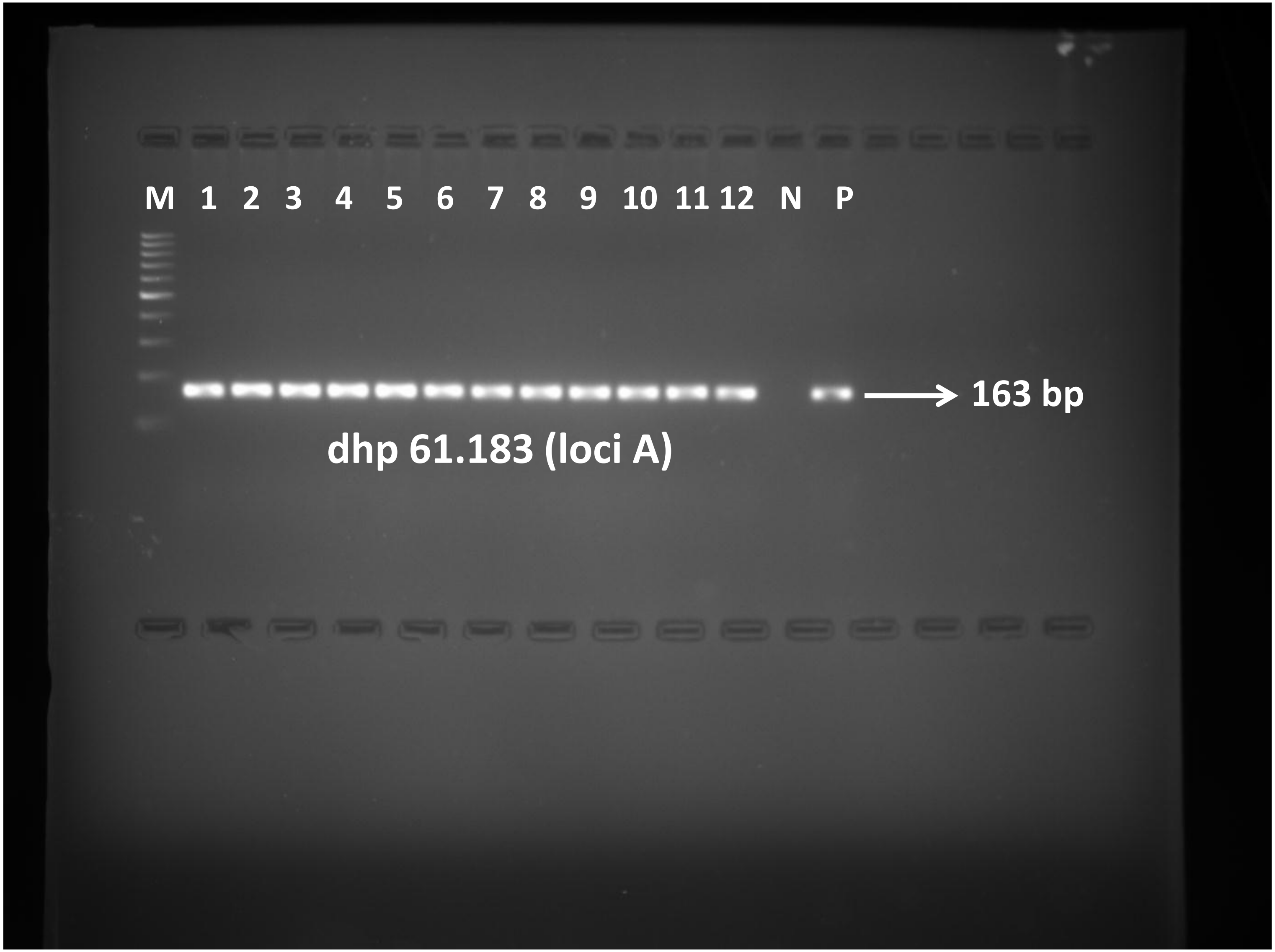

Additional File 3B. PCR for the screening of *Bacillus anthracis* specific loci in the 12 *B. anthracis* DFR.BHE strains 1-12.
(a) dhp 61.183 (loci A)
Lane 1-12, *B. anthracis* DFRL.BHE strains 1-12 along with positive (Lane P: *B. anthracis* BA10) and negative (Lane N: *B. cereus* ATCC 14579) controls. Lane M : 100 bp molecular marker.

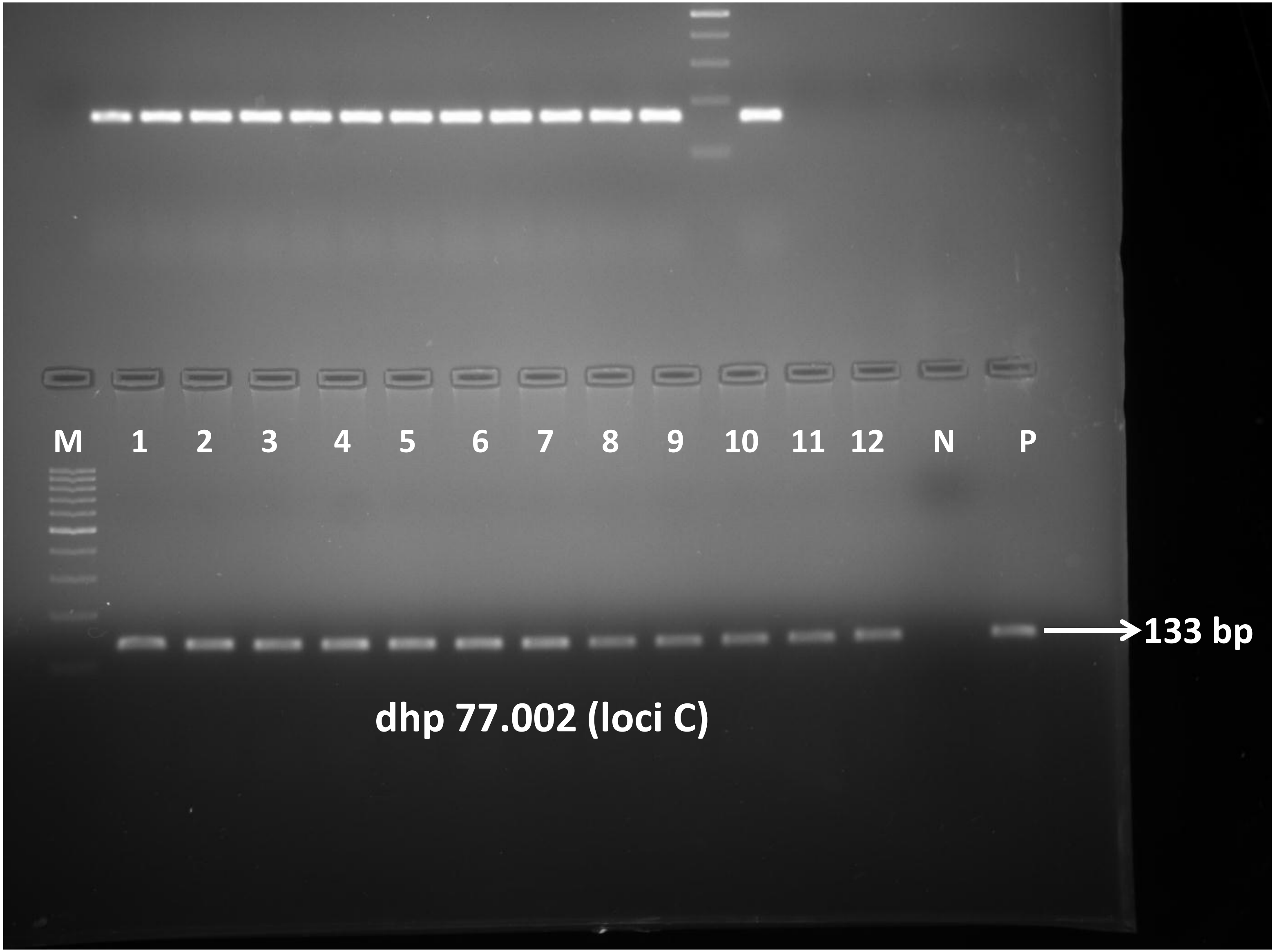

Additional File 3B. PCR for the screening of *Bacillus anthracis* specific loci in the 12 *B. anthracis* DFR.BHE strains 1-12.
(b) dhp 77.002 (loci C)
Lane 1-12, *B. anthracis* DFRL.BHE strains 1-12 along with positive (Lane P: *B. anthracis* BA10) and negative (Lane N: *B. cereus* ATCC 14579) controls. Lane M : 100 bp molecular marker.

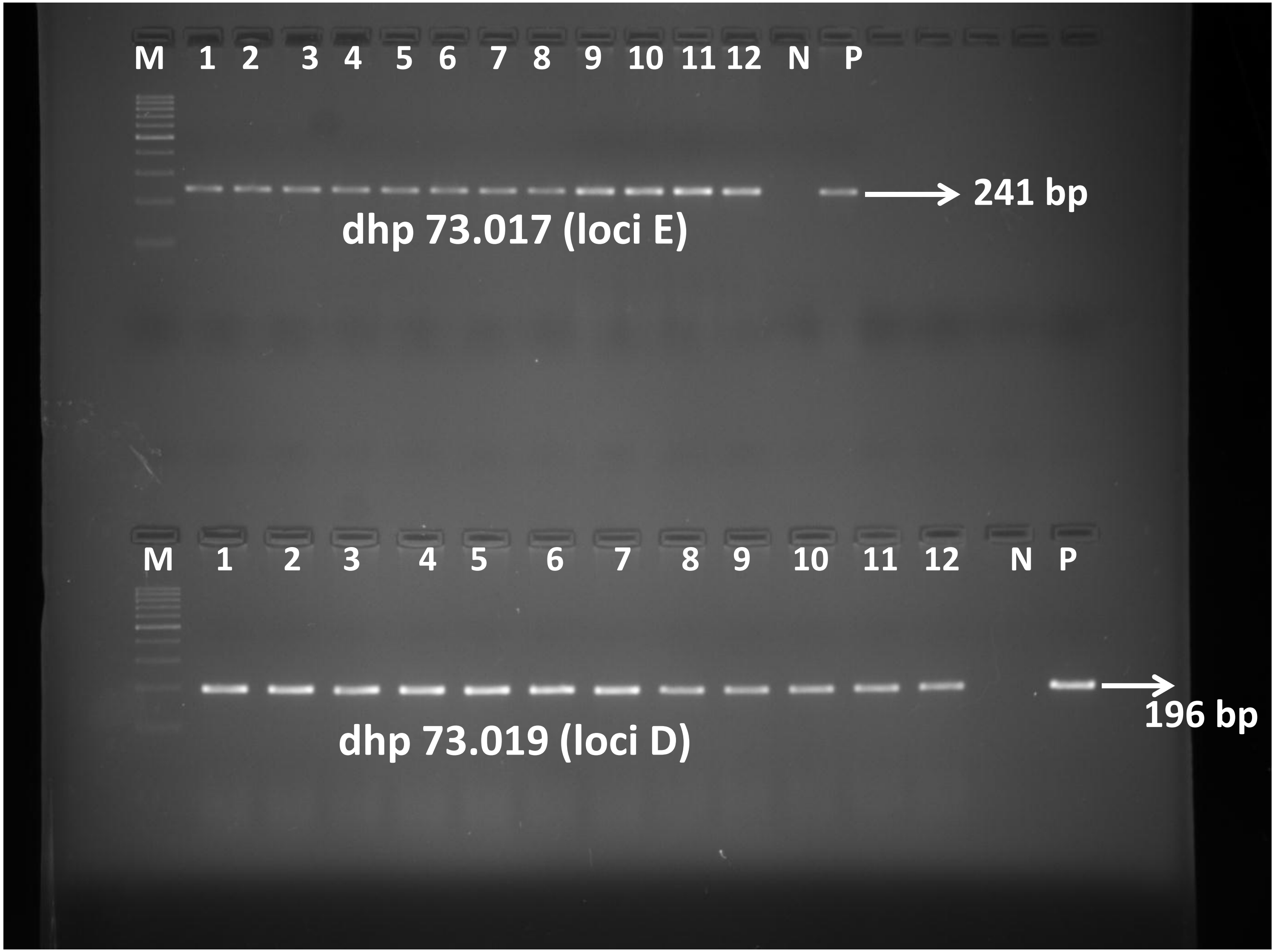

Additional File 3B . PCR for the screening of *Bacillus anthracis* specific loci in the 12 *B. anthracis* DFR.BHE strains 1-12.
(d) dhp 73.017 (loci E), (c) dhp 73.019 (loci D),
Lane 1-12, *B. anthracis* DFRL.BHE strains 1-12 along with positive (Lane P: *B. anthracis* BA10) and negative (Lane N: *B. cereus* ATCC 14579) controls. Lane M : 100 bp molecular marker.
