# Supplementary material for: Molecular characterization of B. anthracis isolates from the anthrax outbreak among cattle in Karnataka, India
Source: BMC Microbiol. 2020 Jul 31;20:232. doi: 10.1186/s12866-020-01917-1 (PMC7394690; doi:10.1186/s12866-020-01917-1)
Supplement: Supplementary file 4 — Additional file 4. List of bacterial cultures used in the present study. [file 12866_2020_1917_MOESM4_ESM.docx]

| **S.No.** | **Culture Name** | **Organism** | **Source** |
| --- | --- | --- | --- |
|  | DFR.BHE 1 | *B. anthracis* | DFRL Repository, Mysore |
|  | DFR.BHE 2 | *B. anthracis* | DFRL Repository, Mysore |
|  | DFR.BHE 3 | *B. anthracis* | DFRL Repository, Mysore |
|  | DFR.BHE 4 | *B. anthracis* | DFRL Repository, Mysore |
|  | DFR.BHE 5 | *B. anthracis* | DFRL Repository, Mysore |
|  | DFR.BHE 6 | *B. anthracis* | DFRL Repository, Mysore |
|  | DFR.BHE 7 | *B. anthracis* | DFRL Repository, Mysore |
|  | DFR.BHE 8 | *B. anthracis* | DFRL Repository, Mysore |
|  | DFR.BHE 9 | *B. anthracis* | DFRL Repository, Mysore |
|  | DFR.BHE 10 | *B. anthracis* | DFRL Repository, Mysore |
|  | DFR.BHE 11 | *B. anthracis* | DFRL Repository, Mysore |
|  | DFR.BHE 12 | *B. anthracis* | DFRL Repository, Mysore |
|  | BA10 | *B. anthracis* | DFRL Repository, Mysore |
|  | Bc. ATCC 14579 | *B. cereus* | DFRL Repository, Mysore |

**Additional File 4. List of bacterial cultures used in the present study.**
