# Supplementary material for: Molecular characterization of B. anthracis isolates from the anthrax outbreak among cattle in Karnataka, India
Source: BMC Microbiol. 2020 Jul 31;20:232. doi: 10.1186/s12866-020-01917-1 (PMC7394690; doi:10.1186/s12866-020-01917-1)
Supplement: Supplementary file 5 — Additional file 5. List of primers and their amplicons size used in the present study. [file 12866_2020_1917_MOESM5_ESM.docx]

| **Gene**  **Additional File 5: List of primers and their amplicons size used in the present study.** | **Forward (5’-3’)** | **Reverse (5’-3’)** | **Amplicon Size** |
| --- | --- | --- | --- |
| **For amplification** **of Protective Antigen gene (This study)** | | |  |
| pag  (PA- F and PA-R) | CAGGAGAACCGGTTATTA | TCTTGATCCCGTTGGTAC | 2158 bp |
| **For amplification** **of 16S RNA gene (Brosius et al. [54])** | | | |
| 16SrRNA  (16S-27F and 16S-1488R) | AGAGTTTGATCATGGCTCAG | ACGGTTACCTTGTTACGACT | 1.5 kb |
| **For amplification** ***B. anthracis*** **prophage genes (Sozhamannan et al. [8])** | | | |
| LambdaBa01 | AACAAATTCAGTTGCGCTTCC | CGATGAAGTGACGAGCATCAT | 221 bp |
| LambdaBa02 | TCACTTGCCAGTCTTGACCTTG | GTTTCGGCAGTGAAGATGGAC | 233 bp |
| LambdaBa03 | ACGTTACCCCTATTTCCGAAGC | CAGAAGTTAAGCCAGTACGGCC | 189 bp |
| LambdaBa04 | CCAGTTGAATCCAGAACAAACG | GCTTCCATGCTCGTTACAATTC | 157 bp |
| **For amplification** **of *B. anthracis* specific loci marker (Radnedge et al. [7])** | | | |
| dhp 61.183 (loci A) | GAAGGACGATACAGACATTTATTGG | ACCGCAAGTTGAATAGCAAG | 163 bp |
| dhp 77.002 (loci C) | TGATATTTATGACCAAGATTCAATATACG | GCCATAGCTCAAGGTCAATAGG | 133 bp |
| dhp 73.019 (loci D) | AAAGGCGGTTTAGAATTTGG | TGCTGCTCTTTACCCATGC | 196 bp |
| dhp 73.017 (loci E) | TGTAAATGAACGCCTTGACC | CCGACTCCTTCTATCAATTCC | 241 bp |
| **For amplification** **of *B. anthracis* specific canSNP loci (Van Ert et al. [9])** | | | |
| A.Br.001 | GTGGTAAGGCAAGCGGAAC | ACGGTTTCCCTTTATCATCG | 76 bp |
| A.Br.002 | GCAGAAGGAGCAAGTAATGTTATAGGT | CCTAAAATCGATAAAGCGACTGC | 62 bp |
| A.Br.003 | AAAGGAATTTAGATTTTCGTGTCG | ATAAAAACCTCCTTTTTCTACCTCA | 58 bp |
| A.Br.004 | ATCGCCGTCATACTTTGGAA | GGAATTGGTGGAGCTATGGA | 53 bp |
| A.Br.006 | GCGTTTTTAAGTTCATCATACCC | ATGTTGTTGATCATTCCATCG | 54 bp |
| A.Br.007 | TTACAAGGTGGTAGTATTCGAGCTG | TTGGTAACGAGACGATAAACTGAA | 67 bp |
| A.Br.008 | CCAAACGGTGAAAAAGTTACAAA | GCAACTACGCTATACGTTTTAGATGG | 67 bp |
| A.Br.009 | AATCGGCCACTGTTTTTGAAC | AGGTATATTAACTGCGGATGATGC | 55 bp |
| B.Br.001 | GCACGGTCATAAAAGAAATCG | TGTTCAAAAGGTTCGGATATGA | 75 bp |
| B.Br.002 | GCACCTTCTGTGTTCGTTGTT | TTCACCGAATGGAGGAGAAG | 68 bp |
| B.Br.003 | ATTCGCATAGAAGCAGATGAGC | TCAAGTTCATAACGAACCATAACG | 62 bp |
| B.Br.004 | TGCTTGGGTAACCTTCTTTACTT | AGAATAAAATGAAGATAATGACAAACG | 62 bp |
| A/B.Br.001 | ATTCCAATCGCTGCACTCTT | CCCCGATAATTTTCACAAAGC | 59 bp |
| **For Sequencing (This study)** | | |  |
| M13 | GTAAAACGACGGCCAGT | CAGGAAACAGCTATGAC | - |
| PA F1 | GTTGTACTGGACCGATTC | - | - |
| PA F2 | GTTCTTTGATATTGGTGGGAG | - | - |
