# Supplementary material for: Molecular characterization of B. anthracis isolates from the anthrax outbreak among cattle in Karnataka, India
Source: BMC Microbiol. 2020 Jul 31;20:232. doi: 10.1186/s12866-020-01917-1 (PMC7394690; doi:10.1186/s12866-020-01917-1)
Supplement: Supplementary file 6 — Additional file 6. List of strains and their accession numbers used for multiple sequence alignment of 16S rDNA gene and pag gene in the present study. [file 12866_2020_1917_MOESM6_ESM.docx]

| **Name of strains used for 16S rDNA sequence analysis**  **Additional File 6: List of *Bacillus cereus sensu stricto* strains and their accession numbers used for multiple sequence alignment of 16S rDNA gene and *pag* gene in the present study.** | **Accession No.** |
| --- | --- |
| *B. mycoides* strain 10206 | AF155957.1 |
| *B. cereus strain* H1439 | AY138270.1 |
| *B. cereus strain* G8639 | AY138271.1 |
| *B. thuringiensis strain* 4Q2811 | AF155954.1 |
| *B. cereus strain* Delaporte | AF155958.1 |
| *B. anthracis strain* 2000031650 | AY138379.1 |
| *B. anthracis strain* 2000031651 | AY138372.1 |
| *B. anthracis strain* 2000031653 | AY138373.1 |
| *B. anthracis strain* 2000031656 | AY138375.1 |
| *B. anthracis strain* AMES4 | CP009981.1 |
| *B. anthracis strain* 2001039370 | AY138291.1 |
| *B. anthracis strain* 2002007651 | AY138355.1 |
| *B. anthracis strain* 2002007650 | AY138356.1 |
| *B. anthracis strain* 2002007649 | AY138357.1 |
| *B. anthracis strain* 2000031666 | AY138352.1 |
| *B. anthracis strain* 2000031078 | AY138351.1 |
| *B. anthracis strain*Vollum | AF290553.1 |
| *B. anthracis strain* Ames3 | CP009981.1 |
| *B. anthracis strain* Delta Ames | CP008752.1 |
| *B. anthracis* | AE017334.2 |
| *B. cereus strain* 2000031491 | AY138276.1 |
| *B. cereus strain* 2000031503 | AY138279.1 |
| *B. cereus strain* G3317 | AY138278.1 |
| *B. cereus strain* 2000031498 | AY138274.1 |
| *B. cereus strain* LRN | JX966388.1 |
| *B. cereus strain* 9620 | AF155952.1 |
| *B. cereus strain* ATCC11778 | CP098263 |
| *B. cereus* | CP985262 |
| *B. cereus strain* ATCC14579genBank | CP762413 |
| *B. cereus strain* ATCC31293 | CP092452 |
| *B. thuringiensis strain* 2000031482 | AY138290.1 |
| *B. thuringiensis strain* 2000031485 | AY138289.1 |
| *B. thuringiensis strain* 2000031494 | AY138288.1 |
| *B. thuringiensis strain* 2000031496 | AY138287.1 |
| *B. thuringiensis strain* 2000031508 | AY138286.1 |
| *B. thuringiensis strain* 2000031509 | AY138285.1 |
| *B. thuringiensis strain* 2002007400 | AY138283.1 |
| *B. thuringiensis strain* 2002017401 | AY138284.1 |
| *B. thuringiensis strain* 2000032755 | AY138282.1 |
| *B. thuringiensis strain* 2000032757 | AY138280.1 |
| *B. thuringiensis strain* 2000032756 | AY138281.1 |
| *B. thuringiensis strain* ATCC33679 | JF749283.1 |
| *B. thuringiensis strain* B8 | AF155955.1 |
| *B. cereus strain* 2000031486 | AY138272.1 |
| *B. cereus strain* G9667 | AY138273.1 |
| *B. cereus strain* ATCC43881 | AF290550.1 |
| *B. cereus strain* 2000031513 | AY138279.1 |
| *B. thuringiensis strain* HS18.1 | CP012099.1 |
| *B. thuringiensis strain* MYBT.18246 | CP015350.1 |
| *B. cereus strain* MLY1.0 | CP024655.1 |
| *B. thuringiensis strain* DAR81934 | NZ_CM001804.1 |
| *B. anthracis strain* Sterne | KP942847.1 |
| *B. anthracis strain* PAK.1 | CP009325.1 |
| *B. anthracis strain* BA1015 | CP009544.1 |
| *B. cereus strain* FM1 | CP009544.1 |
| *B. cereus strain* G9241 | AY425946.1 |
| *B. cereus strain* 03BB102 | CP009318.1 |
| *B. cereus strain* E33L | CP009968.1 |
| *B. thuringiensis* serovar konkukian strain 97-27 | NC_005957.1 |
| *B. thuringiensis strain* HD682 | NZ_CP009720.1 |
| *B. thuringiensis strain* Al- Hakam | CP000485.1 |
| *B. thuringiensis strain* HD1011 | CP009335.1 |
| *B. thuringiensis strain* HD571 | CP009600.1 |
| *B. anthracis strain*  H9401 | NC_017729.1 |
| *B. anthracis strain* Tangail | CP015779.1 |
| *B. anthracis strain* CEB9570033 | AM747220.1 |
| *B. anthracis strain* L22 | LN890018 |
| *B. anthracis strain* Ames1 | AE016879.1 |
| *B. cereus strain* CC-1 | CP023179.1 |
| *B. thuringiensis strain* serovar BGSC4Y1 | CP010577 |
| *B. thuringiensis strain* serovar finitimusYBT-020 | CP002508.1 |
| *B. thuringiensis strain* 15426 | CP020721 |
| *B. thuringiensis strain* serovar BGSC4AW1 | CP010578 |
| *B. thuringiensis strain* serovar pondicheriensis BGSC4BA1 | CP010576 |
| *B. thuringiensis strain* serovar pulsiensis BGSC4CC1 | CP010574 |
| *B. thuringiensis strain* KNU-07 | NZ_CP016590.1 |
| *B. thuringiensis strain* BT185 | CP014282.1 |
| *B. cereus strain* FORC-024 | NZ_CP012691.1 |
| *B. cereus strain* G9842 | NC_011772.1 |
| *B. cereus strain* ATCC14579 | AF290547.1 |
| *B. cereus strain* B4264 | CP001176.1 |
| *B. cereus strain* A1 | CP015727.1 |
| *B. cereus strain* FORC-005 | NZ_CP009686.1 |
| *B. cereus strain* FORC-013 | NZ_CP011145.1 |
| *B. cereus strain* HN001 | CP011155.1 |
| *B. cereus strain* FORC021 | CP014486.1 |
| *B. cereus strain* M13 | CP016360.1 |
| *B. cereus strain* FORC60 | NZ_CP020383.1 |
| *B. anthracis strain* Canadian.bison | CP010322.1 |
| *B. anthracis strain* OhioACB | CP009341.1 |
| *B. anthracis strain* 2002013094 | NZ_CP009902.1 |
| *B. anthracis strain* Ames2 | AE016879.1 |
| *B. anthracis strain* Vollum.1B | NZ_CP009328.1 |
| *B. anthracis strain* SK-102 | CP009464.1 |
| *B. anthracis strain* Pasteur | CP009476.1 |
| *B. anthracis strain* 1035 | CP009700 |
| *B. anthracis strain* RA3 | CP009697 |
| *B. anthracis strain* V770-NP-1R | CP009598 |
| *B. cereus strain* 03BB108 | CP009641 |
| *B. cereus strain* 3a | CP009596 |
| *B. cereus strain* S2-8 | CP009605 |
| *B. anthracis strain* FDAARGOS | CP022044 |
| *B. anthracis strain* JPR-02 | HE716942 |
| *B. anthracis strain* Ames Ancestor | AE017334 |
| *B. anthracis strain* Ames A0462 | CP010858 |
| *B. anthracis strain* A1144 | CP010852 |
| *B. anthracis strain* Stendal | CP014179 |
| *B. anthracis strain* Han | CP008854 |
| *B. anthracis strain* Cvac02 | CP008853 |
| *B. anthracis strain* A0157 | CP010856 |
| *B. anthracis strain* Pollino | CP010813 |
| *B. anthracis strain* 52-G | CP010855 |
| *B. anthracis strain* 9080-G | CP010851 |
| *B. anthracis strain* A0248 | CP001598 |
| *B. anthracis strain* A16R | CP001974 |
| *B. anthracis strain* London-499 | CP029805 |
| *B. anthracis strain* SVA11 | CP006742 |
| *B. anthracis strain* PR07 | CP012724 |
| *B. anthracis strain* SPV842 | CP019588 |
| *B. cereus strain* biovar *anthracis* strain CI | CP001746 |
| *B. anthracis strain* K3 | CP009331.1 |
| *B. anthracis strain* Turkey32 | CP009315.1 |
| *B. cereus strain* D17 | CP009300.1 |
| *B. cereus strain* ATCC4342 | CP009628.1 |
| *B. thuringiensis strain* HD1002 | CP009351.1 |
| *B. thuringiensis strain* serovar kurstaki strain HD-1 | CP004870.1 |
| *B. thuringiensis strain* serovar morrisoni BGSC4AA1 | CP010577.1 |
| *B. mycoides strain* ATCC6462 | NR_115993.1 |
| *B. anthracis strain* L10 | LN890006.1 |
| *B. anthracis strain* AB11D | AM062681.1 |
| *B. anthracis strain* CDC684 | CP00121 |
| *B. anthracis strain* Tyrol4675 | CP018903.1 |
| *B. anthracis strain* L23 | LN890019.1 |
| *B. cereus strain* 03BB87 | CP009941.1 |
| *B. cereus strain* NJ-W | CP012483.1 |
| *B. cereus strain* CMCCP0021 | NZ_CP011152.1 |
| *B. cereus strain* CMCCP0011 | NZ_CP011153.1 |
| *B. cereus strain* M3 | CP016316.1 |
| *B. cereus strain* ATCC10987 | AE017194.1 |
| *B. cereus strain* BC-AK | CP020937.1 |
| *B. cereus strain* FRI-35 | CP003747.1 |
| *B. cereus strain* AH820 | CP001283.1 |
| *B. cereus strain* Q1 | GQ280380.1 |
| *B. cereus strain* AH187 | CP001177.1 |
| *B. cereus strain* NC7401 | AP007209.1 |
| *B. cereus strain* K8 | CP016595.1 |
| *B. cereus strain* TG1-6 | CP026678.1 |
| *B. thuringiensis strain* YGd22-03 | CP019230.1 |
| *B. thuringiensis strain* Bt407 | CP003889.1 |
| *B. thuringiensis strain* YBT-1518 | NC_022873.1 |
| *B. thuringiensis strain* serovar chinensis CT-43 | CP001907.1 |
| *B. thuringiensis strain* HD-789 | CP003763.1 |
| *B. thuringiensis strain* serovar BGSC4C1 | AY224387.1 |
| *B. thuringiensis strain* BT18247 | CP015250.1 |
| *B. thuringiensis strain* ATCC10792 | CP020754.1 |
| *B. thuringiensis strain* L-7601 | CP020002.1 |
| *B. thuringiensis strain* CTC | CP013274.1 |
| *B. thuringiensis* serovar kurstaki strain YBT-1520 | CP004858.1 |
| *B. thuringiensis* serovar indiana HD521 | CP010106 |
| *B. thuringiensis strain* HD12 | CP014847.1 |
| *B. thuringiensis* serovar galleriae HD-29 | CP010089.1 |
| *B. thuringiensis* serovar kurstaki HD1 | CP010005.1 |
| *B. thuringiensis* serovar kurstaki strain HD73 | CP004069.1 |
| *B. thuringiensis strain* BMB171 | CP001903.1 |
| *B. thuringiensis strain* YWC2-8 | CP013055.1 |
| *B. thuringiensis strain* BC601 | CP015150.1 |
| *B. mycoides strain* Gnyt1 | KY887028.1 |
| *B. mycoides strain* DSM2048 | LS998020.1 |
| *B. mycoides strain* AH603 | NZ_CM000737.1 |
| *B. mycoides strain* AH621 | NZ_CM000719.1 |
| *B. anthracis* strain Sterne | CP009540.1 |
| *B. anthracis* strain Ohio ACB | CP009340.1 |
| *B. anthracis* strain BFV | CP007703.1 |
| *B. anthracis* strain A16 | CP001971.2 |
| *B. anthracis* strain A0248 | CP001599.1 |
| *B. anthracis* strain A2012 | AE011190.1 |
| *B. anthracis* strain Ames Ancestor | CP009980.1 |
| *B. anthracis* strain SPV842_15 | CP019589.1 |
| *B. anthracis* strain FDAARGOS-341 | CP022045.2 |
| *B. anthracis* strain Shikan-NIIID | AP014834.1 |
| *B. anthracis* strain HYU01 | CP008847.1 |
| *B. anthracis* strain SK-102 | CP009463.1 |
| *B. anthracis* strain Vollum | CP007665.1 |
| *B. anthracis* strain CDC684 | CP001216.1 |
| *B. anthracis* strain Vollum 1B | CP009327.1 |
| *B. anthracis* strain BA1035 | CP009699.1 |
| *B. anthracis* strain SVA11 | CP006743.1 |
| *B. anthracis* strain Turkey32 | CP009316.1 |
| *B. anthracis* strain BA1015 | CP009543.1 |
| *B. anthracis* strain Canadian_Bison | CP010321.1 |
| *B. anthracis* strain K3 | CP009330.1 |
| *B. anthracis* strain PAK-1 | CP009324.1 |
| *B. anthracis* strain RA3 | CP009696.1 |
| *B. anthracis* strain H9401 | CP002092.1 |
| *B. anthracis* strain V770-NP-1R | CP009597.1 |
| *B. anthracis* strain London_499 | CP029806.1 |
| *B. anthracis* strain 2002013094 | CP009901.1 |
| *B. cereus* strain 03BB102 | CP001406.1 |
| *B. cereus* strain 03BB87 | CP009941.1 |
| *B. cereus* biovar *anthracis* strain CI | CP001747.1 |
| *B. cereus* strain G9241 | DQ889680.1 |
| *B. cereus* strain BC-AK | CP020940.1 |
